# Common metabolic, environmental, and molecular mechanisms underlying neurodevelopmental and neurodegenerative disorders

**DOI:** 10.3389/fneur.2026.1860052

**Published:** 2026-09-04

**Authors:** Ana Sofía Vallés, Francisco J. Barrantes

**Affiliations:** 1 Laboratorio de Nutrición y Neurodesarrollo, Instituto de Investigaciones Bioquímicas de Bahía Blanca, BByF-UNS-CONICET, Bahía Blanca, Argentina; 2 Molecular Neurobiology Division, Biomedical Research Institute, UCA-CONICET, Buenos Aires, Argentina

**Keywords:** Alzheimer disease, neurodegenerative diseases, neurodevelopmental disorders, two-hit hypothesis, α7 nicotinic acetylcholine receptor

## Abstract

Several neurodevelopmental disorders and neurodegenerative diseases share common pathogenic mechanisms that unfold across the lifespan, blurring the distinction between the two nosological entities. Environmental factors, particularly those shaping metabolic health during critical developmental time windows, have emerged as key modulators of long-term brain liabilities. This review critically evaluates the experimental, epidemiological, and mechanistic evidence linking early-life metabolic and environmental insults with the establishment of latent vulnerability. We propose that this status may remain clinically silent for decades until activated by aging and cumulative stressors, ultimately leading to neurodegeneration. Specifically, we analyze how early metabolic alterations disrupt mitochondrial function, redox signaling, synaptogenesis, and glial programming, particularly in microglia, thereby shaping long-term neuroinflammatory tone and dysfunctional neural circuit maturation. The review highlights the role of the α7 nicotinic acetylcholine receptor (α7nAChR) as a strategic molecular bridge onto which metabolic and inflammatory signals converge. We further discuss how early dysregulatory stressors manifest later in life as dysmetabolism, vascular impairment, and defective energy sensing, all of which accelerate neurodegenerative pathophysiological processes. This life-course perspective reframes these disorders as a continuum and highlights critical prophylactic and/or therapeutic opportunities through early nutritional, metabolic, and environmental interventions.

## Introduction

Traditionally, neurodevelopmental disorders and neurodegenerative diseases have been conceptualized as temporally and mechanistically distinct categories of pathological processes. However, the two categories comprise heterogeneous collections of pathological affectation of neurons in the central or peripheral nervous systems. Focus on their age of onset -early in the former and late in the latter- has often obscured accumulating evidence suggesting that the two types of disorders share convergent molecular, cellular, and metabolic pathophysiological pathways (1, 2). Conditions such as autism spectrum disorder, intellectual disability, Alzheimer disease, and Parkinson disease display overlapping signatures of mitochondrial dysfunction, oxidative stress, neuroinflammation, and synaptic dysregulation (3, 4).

Current evidence points to a multifaceted, complex interaction between genetic pre-disposition and environmental triggers that converge, leading to systemic metabolic and immune disruptions that significantly impact the central nervous system (5). A good example of the neurodevelopmental/neurodegenerative continuum is the correlation between autism spectrum disorder and dementias such as Alzheimer disease or frontotemporal dementia; far from being unrelated nosological entities, they in fact share overlapping genetic risk profiles and convergent pathophysiological pathways, often exacerbated by similar environmental stressors. Epidemiological patterns further reinforce this interconnectedness. Families with a history of Alzheimer disease show a higher incidence of autism spectrum disorder in younger generations (6). Moreover, the risk of cognitive decline in the autistic population is notably elevated; adults with autism spectrum disorder under the age of 65 are approximately 2.6 times more likely to be diagnosed with early-onset dementia than the average, neurotypical population (7). However, these findings underscore a statistical association rather than a linear, deterministic progression from neurodevelopmental traits to neurodegeneration. Furthermore, the clinical heterogeneity inherent to autism spectrum disorder suggests that this vulnerability is far from uniform, varying significantly across individuals. Interpreting this link requires careful consideration of critical confounding factors, including high rates of metabolic, immunological, and psychiatric comorbidities, as well as the long-term impact of chronic stress and systemic healthcare disparities faced by the autistic population along their lifespan.

Environmental exposures, particularly those impacting metabolic health, have emerged as critical modulators of brain vulnerability (8, 9). Specifically, factors such as poor nutritional quality, maternal metabolic syndrome, obesity, and insulin resistance during development exert long-lasting effects on neural structure and function (10, 11). Among these, maternal immune activation represents a major prenatal environmental factor through which inflammatory and metabolic disturbances can induce persistent alterations in immune programming, synaptic development, and neuroinflammatory tone, thereby increasing long-term brain vulnerability (12, 13). These observations underline the need to reconceptualize brain disorders as life-course conditions, shaped by early programming and subsequent cumulative insults.

In this review, we describe an integrative two-hit framework that unifies neurodevelopmental and neurodegenerative processes within a shared etiopathogenic continuum. The first hit occurs during sensitive developmental time windows in which metabolic and environmental insults disrupt brain maturation and pathologically “prime” neuronal circuits. The second hit ensues later in life, driven by aging, additional metabolic stressors, and vascular and inflammatory changes that precipitate neurodegenerative pathology. Hence, this model reframes neurodegenerative and neurodevelopmental diseases as points along a shared neuropathological continuum rather than discrete, unrelated categories. It provides a mechanistic basis for understanding how early-life metabolic and environmental perturbations may contribute to increased susceptibility to neurodegeneration later in life. Accordingly, it supports a shift from late-stage symptomatic treatment to preventive strategies that preserve metabolic health during critical developmental windows.

Within this framework, molecular systems that regulate energy sensing, neuroinflammation, and synaptic plasticity, such as microglial metabolic programming and cholinergic signaling, emerge as critical integrators of environmental risks across the lifespan. The idiopathic, late-onset form of Alzheimer disease (LOAD), which accounts for 95% of the presentations, is used here as a paradigmatic example in which such factors converge together with genetics and comorbidities in a complex and multifactorial manner to disrupt homeostasis. As developed in the present review, such an etiopathogenic framework applies to a broader spectrum of conditions sharing metabolic and neuroimmune vulnerability.

## Methodology

### Review methodology

This narrative review is based on a comprehensive search of the peer-reviewed literature conducted primarily using PubMed/MEDLINE, complemented by Google Scholar to identify additional relevant articles and recent publications. The search focused on articles published between 2016 and 2026, while earlier seminal publications were included when necessary to provide historical context or support key mechanistic concepts. Search terms included combinations of neurodevelopment, neurodegeneration, maternal metabolic syndrome, metabolic programming, developmental origins of health and disease, microglia, mitochondrial dysfunction, oxidative stress, neuroinflammation, α7 nicotinic acetylcholine receptor, fructose metabolism, synaptic plasticity, two-hit hypothesis and Alzheimer disease. Original experimental studies, epidemiological investigations, translational studies, and relevant review articles were considered. Particular emphasis was placed on studies providing mechanistic evidence linking early-life metabolic and environmental exposures with long-term alterations in brain development, neuroimmune regulation, and susceptibility to neurodegenerative diseases. References were selected based on scientific relevance, methodological rigor, and their contribution to the integrative life-course framework proposed here. The literature search was updated through April 2026.

## Results

### Early-life programming

Early life represents a period of heightened plasticity and vulnerability during which environmental signals exert a disproportionately strong influence on brain development (14, 15). Nervous system development begins during embryogenesis and suboptimal nutrition, maternal obesity, gestational diabetes, and metabolic syndrome can alter in utero and neonatal metabolic environments, inducing long-term maladaptive neuropathological changes (16–20). Experimental models demonstrate that early metabolic insults induce mitochondrial dysfunction, impaired oxidative phosphorylation, and altered redox signaling in the developing brain (21, 22). These metabolic alterations compromise synaptogenesis, neuronal differentiation, and the refinement of neuronal networks, establishing aberrant connectivity patterns.

Microglia play a central role in these programming mechanisms. As metabolically sensitive immune cells, microglia undergo metabolic reprogramming in response to environmental cues, thereby shaping neuroinflammatory tone, synaptic pruning, and neuronal circuit maturation (23, 24). These early metabolic challenges can lead to the establishment of “primed” microglia, exhibiting a low pro-inflammatory threshold and a state of latent vulnerability that may persist into adulthood (25).

### Latent vulnerability and silent pathology

Early-life metabolic and environmental insults frequently remain clinically silent for decades, yet they leave enduring biological imprints that shape long-term brain vulnerability. This latent state is characterized by persistent alterations in cellular energy sensing, reduced mitochondrial resilience, chronic low-grade neuroinflammation, and increased vascular fragility (26–28). Neurodevelopmental disorders, despite their clinical heterogeneity, increasingly converge on shared mechanisms of synaptic dysfunction. Synapses rely on precisely coordinated molecular machinery whose spatial localization and vectorial specialization (pre-synaptic/post-synaptic), regional brain expression, and temporal activity across developmental stages critically determine functional outcomes (29).

Because many genes coding for synaptic proteins exhibit spatiotemporal pleiotropy, early perturbations may subtly reprogram network architecture without producing immediate clinical manifestations (30). Consequently, latent vulnerability can be understood as a form of “silent pathology,” in which neurodevelopmental alterations remain subclinical but lower the threshold for neurodegeneration when additional metabolic, inflammatory, or vascular insults occur later in life.

Consistent with this concept, emerging clinical and genomic data indicate that disruption of shared synaptic protein networks contributes to a broad spectrum of neurodevelopmental and systemic phenotypes, highlighting the network-based nature of these disorders (30). Epigenetic mechanisms further consolidate this latent state. Early environmental exposures induce stable modifications in chromatin structure and gene expression (31), effectively embedding developmental experience into long-term transcriptional programs. This epigenetic “memory” preserves altered regulatory set points, maintaining the brain in a primed yet vulnerable condition that is poised for reactivation by aging, metabolic stress, or secondary insults (32–34).

### Aging and cumulative stressors

Within the context of latent vulnerability, aging and cumulative metabolic stressors do not act as independent causative factors, but rather as triggers that unmask and amplify pre-existing neurobiological fragility. Aging represents a major biological stressor characterized by declining mitochondrial efficiency, impaired autophagy, vascular dysfunction, and systemic inflammation (35). In individuals previously “programmed” by early metabolic insults, these age-related changes constitute a second hit.

Late-life metabolic stressors such as insulin resistance, dyslipidemia, mid-life obesity sustained into old age, and cardiovascular disease, reactivate the same molecular pathways disrupted during development. These include oxidative stress, lipid dysregulation, impaired synaptic plasticity, and the transition of “primed” microglia into a chronic neuroinflammatory state (36–40). The cumulative interaction of these mechanisms increases the likelihood of clinical neurodegeneration, exemplified by disorders such as Alzheimer and Parkinson diseases.

### Prophylactic and translational implications

Targeting modifiable factors such as maternal and childhood nutrition, metabolic balance, vascular health, and the regulation of neuroinflammatory priming during development may not only reduce neurodevelopmental risk but also mitigate long-term neurodegenerative vulnerability. Accordingly, public health strategies focused on optimizing maternal nutrition, promoting metabolic health throughout early-life, and improving the developmental environment may provide powerful and cost-effective primary interventions by addressing mechanisms that influence brain health across the lifespan.

### α7 Nicotinic acetylcholine receptors as a molecular bridge in the two-hit model

The α7 nicotinic acetylcholine receptors (α7nAChRs) are critical players in the neurodevelopmental-neurodegenerative continuum model, linking early neurodevelopmental programming with long-term vulnerability to neurodegenerative processes (41–43). Due to its high calcium permeability and broad expression across neurons, astrocytes, microglia, and other neuroimmune cells, under physiological conditions, the α7nAChR exerts key regulatory functions in intracellular calcium signaling, synaptogenesis, mitochondrial function, redox balance, and neuroinflammatory responses (44–46). Because α7nAChRs are dynamically regulated throughout development and aging, they function as key integrators of environmental, metabolic, neuroinflammatory, and synaptic signals across the lifespan. Dysregulation of α7nAChR-mediated signaling during sensitive periods may permanently alter such homeostatic set-points, increasing long-term susceptibility to neurodegenerative processes.

### Roles of α7nAChRs at neurodevelopmental stages: initial molecular hit

During brain development, α7nAChR signaling plays a critical role in activity-dependent circuit formation, neuronal differentiation, neurite outgrowth, and synaptic maturation (47). Through calcium-dependent intracellular signaling cascades, α7nAChRs influence neuronal survival, excitatory–inhibitory balance, mitochondrial biogenesis, synaptic plasticity and memory functions, rendering this receptor system particularly sensitive to perturbations during critical developmental windows.

Genetic alterations, epigenetic regulation, or environmentally-induced dysfunction of α7nAChRs have been associated with a spectrum of neurodevelopmental conditions, including autism spectrum disorder, intellectual disability, and attention-related phenotypes (42, 48). Early disruption of α7nAChR signaling may subtly alter synaptic maturation and neural circuit assembly, generating persistent changes that increase susceptibility to later neurological dysfunction.

Disruption of early α7nAChR signaling leads to long-term consequences that cannot be fully accounted for by alteration of neuronal receptors. α7nAChRs are also expressed in astrocytes and microglia, playing a decisive role in translating early-life environmental and metabolic cues into durable changes in brain homeostasis, e.g., in Alzheimer disease (49). As the resident immune cells of the central nervous system, microglia act as central regulators of brain homeostasis across the lifespan, coordinating synaptic development and plasticity, interactions with cholesterol (50), inflammatory signaling, and the clearance of misfolded proteins such as amyloid β (Aβ) and the microtubule-associate protein tau (51–53). Under neurodegenerative conditions, these homeostatic roles become progressively compromised, contributing to chronic neuroinflammation, synaptic failure, and neuronal loss (24). An increasingly recognized driver of microglial dysfunction is metabolic reprogramming induced by environmental and nutritional factors. Among these, fructose metabolism has emerged as a critical but still underappreciated contributor (54).

### Chronic fructose exposure as a population-level metabolic stressor across development

Since its widespread incorporation into the food industry between the 1970s and 1990s, consumption of high-fructose corn syrup has increased nearly 10-fold, largely driven by its frequent use in sweetened beverages and ultra-processed foods (55–57). Despite strong evidence linking excessive sugar intake to non-communicable diseases, global consumption levels remain alarmingly above recommended limits.

Whereas the World Health Organization suggested a limit of 50 g/day of free sugars, recent neurometabolic evidence suggests that for neuroprotective purposes, this intake should ideally remain below 10 g/day to avoid the activation of endogenous fructose production via the polyol pathway in the brain (58–60). However, habitual intake frequently duplicates or even triplicates these values (61–64).

Beyond systemic metabolic consequences, chronic fructose exposure has increasingly recognized effects on brain metabolism and neuroimmune regulation (59, 65, 66). Importantly, exposure to fructose-rich ultra-processed products often begins *in utero* through maternal diet and is reinforced throughout childhood and adolescence (67, 68). Early-life exposure not only shapes dietary preferences and reward-related behaviors but also establishes enduring metabolic and inflammatory trajectories. Notably, fructose does not simply replace other metabolic stressors; it represents a uniquely unregulated neuroactive substrate that bypasses key metabolic checkpoints, thereby amplifying insulin resistance, lipid dysregulation, and microglial metabolic collapse. Thus, chronic fructose exposure represents a sustained metabolic stressor capable of influencing brain development and long-term neuroimmune homeostasis.

### Microglial fructose metabolism and metabolic reprogramming as a source of latent neuroinflammatory vulnerability

Microglia are metabolically sensitive immune cells that integrate environmental, nutritional, and inflammatory signals across the lifespan. Their functional states critically depend on the balance between glycolysis and mitochondrial oxidative phosphorylation (OXPHOS) (69). Chronic fructose exposure disrupts this balance, driving/triggering maladaptive metabolic reprogramming.

Microglia express high levels of the fructose transporter GLUT5 (SLC2A5), which has a high affinity for fructose. Microglia also express ketohexokinase (KHK), the first and rate-limiting enzyme of the fructolysis pathway, suggesting that they are metabolically equipped to utilize fructose. Both GLUT5 and KHK are upregulated in experimental models of Alzheimer disease and in microglia exposed to Aβ, indicating that fructose metabolism is actively engaged under neurodegenerative conditions (69, 70). However, a direct characterization of fructose metabolism in human microglia *in vivo* remains a key gap in our knowledge, highlighting the urgent need for translational imaging and metabolic profiling approaches.

Chronic exogenous fructose exposure or endogenous fructose production via the polyol pathway induce a glycolytic shift, suppression of mitochondrial OXPHOS, elevated reactive oxygen species production, and intracellular energy depletion. This metabolic collapse leads to lipid droplet accumulation, a phenotype known as Lipid-Laden Microglia (LLM) (71, 72). Under LLM conditions, phagocytic capacity is impaired, and a sustained pro-inflammatory release ensues, mirroring some of the dysfunctional states observed in Alzheimer disease (73, 74).

Genetic or pharmacological inhibition of KHK reduces microglial mitochondrial and oxidative stress and rescues cognitive performance in animal models of type II diabetes (69, 70). Similarly, targeting the microglia-enriched fructose transporter GLUT5 reverses fructose-induced metabolic alterations, dampens inflammatory activation, and restores phagocytic capacity (68). Deletion of Slc2a5 completely prevents microglial metabolic and functional impairments induced by high-fructose diets in neonatal models (68, 75). An additional therapeutic strategy involves inhibition of the polyol pathway, which converts glucose into sorbitol and fructose via aldose reductase (76, 77). Since endogenous fructose production is elevated in Alzheimer disease brains and may precede clinical symptoms, suppressing this pathway could limit pathological fructose accumulation and delay disease progression (59).

Susceptibility to fructose-driven microglial reprogramming exhibits pronounced sexual dimorphism. Female microglia show higher GLUT5 expression and greater metabolic vulnerability with aging and Alzheimer disease, particularly in carriers of the *APOE*ε*4* allele (69, 78, 79). This sex-dependent sensitivity provides a mechanistic link between metabolic stress, microglial dysfunction, and the higher neurodegenerative risk observed in women.

Fructose-driven microglial metabolic reprogramming may establish a persistent state of neuroinflammatory vulnerability in which metabolically altered microglia exhibit an enhanced response to subsequent stressors. Together with α7nAChRs-mediated cholinergic signaling, these pathways integrate metabolic, inflammatory, and synaptic homeostasis providing a mechanistic link between early metabolic disturbances and increased susceptibility to neurodegeneration (44, 80).

### Aging, metabolic stress, and α7nAChR dysfunction: converging pathways to neurodegeneration

Aging-associated metabolic stress intersects with microglial dysfunction and α7nAChR signaling (81, 82). Lipid dysregulation, cholesterol accumulation, insulin resistance, and impaired energy metabolism progressively compromise α7nAChR-mediated cholinergic anti-inflammatory signaling in neurons and glia (83–85). Dysregulation of cholesterol-rich membrane domains alters α7nAChR localization and function (86), while chronic inflammation and amyloid exposure further impair receptor signaling (49). In microglia, reduced α7nAChR activity hampers the resolution of micro-inflammatory status, sustains cytokine production, and reinforces metabolic dysfunction, creating a vicious cycle of neuroinflammation and energy failure (84, 87, 88).

A growing body of evidence indicates that alterations in cholesterol homeostasis in glial cells represent a critical mechanistic link between early metabolic vulnerability and late-life neurodegeneration (89–92). In astrocytes and microglia cells expressing the ApoE4 isoform, lipid metabolic programs are extremely dysregulated, leading to increased *de novo* cholesterol synthesis despite intracellular cholesterol sequestration (93–95). This maladaptive state is thought to initiate glia-driven metabolic stress, priming neural circuits for degeneration along aging.

Astrocyte-derived cholesterol plays a central role in the spatial organization of neuronal membranes, particularly in cholesterol-rich microdomains that regulate amyloid precursor protein (APP) trafficking and processing (96). Excess cholesterol promotes the localization of APP within these domains, facilitating its interaction with secretases and favoring amyloidogenic processing (50, 96, 97). Experimental suppression of astrocytic cholesterol biosynthesis reduces amyloid and tau pathology *in vivo*, supporting a causal role for glial lipid metabolism in the emergence of classical Alzheimer disease neuropathological hallmarks (96, 98). Thus, cholesterol dysregulation constitutes a latent metabolic vulnerability that may remain clinically silent until additional stressors act as a second hit.

Beyond amyloidogenesis, cholesterol-dependent membrane remodeling critically impairs neuronal energy metabolism (99, 100). Under conditions of lipid excess, insulin receptors, which regulate synaptic plasticity and cognitive function, are redistributed into cholesterol-rich membrane domains, where they become functionally impaired and prone to internalization (101–103). This change results in neuronal insulin resistance, reduced glucose uptake, and compromised ATP production. Such deficits align with the neuroenergetic hypothesis of Alzheimer disease, in which chronic or intermittent energy failure contributes to synaptic dysfunction and neuronal loss (104, 105).

*In vivo* studies using FDG-PET have consistently demonstrated early and region-specific reductions in cerebral glucose uptake in Alzheimer disease and in cognitively normal individuals carrying genetic risk factors (106–108). These findings indicate that metabolic failure precedes overt neurodegeneration and may reflect the delayed manifestation of earlier metabolic and lipid disturbances. Similarly, patients with autism spectrum disorders also have decreased brain glucose metabolism, and their left brain regions appear to be more severely affected than those on the right (109). This glucose hypometabolism may thus precede and potentially contribute to neurodegeneration (110–112).

Aging-associated vascular dysfunction, dyslipidemia, and hyperglycemia further exacerbate this energetic deficit by limiting glucose delivery and transport, as evidenced by reduced expression of GLUT1 and GLUT3 in the brains of Alzheimer disease individuals (113) and mouse models of autism spectrum disorders (114).

Recent evidence further supports the central role of bioenergetic dysfunction in neurodegeneration. Pharmacological terazosin-induced activation of glycolysis through phosphoglycerate kinase 1 (PGK1) in an Alzheimer disease murine model has been shown to restore ATP production and improve cognitive function in a sex-dependent manner (115). These findings highlight energy metabolism as a critical node linking metabolic dysfunction, neuroinflammation, and neuronal resilience.

While most evidence derives from studies on sporadic LOAD, similar mechanisms may be relevant in early-onset genetic forms, in which mutations lead to mitochondrial dysfunction and energy imbalance (116, 117). In this context, fructose metabolism may represent a shared downstream pathway linking metabolic stress to neurodegenerative processes across the Alzheimer disease spectrum.

Metabolic stress intersects with neuroinflammatory signaling through microglial activation and α7nAChR dysfunction (84, 118, 119). α7nAChRs are expressed in neurons, astrocytes, and microglia, where they modulate cholinergic anti-inflammatory pathways, metabolic sensing, and synaptic integrity. Cholesterol-rich membrane environments influence α7nAChR localization and modulate its function, whereas chronic inflammation and amyloid exposure disrupt α7nAChR-mediated signaling (120–125). In microglia, this impairment weakens the resolution of inflammation, promotes sustained cytokine release, and further alters lipid metabolism, creating a self-reinforcing cycle.

Collectively, these findings suggest that early disturbances in lipid metabolism, insulin signaling, or α7nAChR–mediated neuroimmune regulation establish a state of concealed vulnerability. During aging, cumulative metabolic stress, inflammatory burden, and declining compensatory capacity act as a second hit, transforming silent pathology into overt neurodegeneration. These converging factors provide a unified molecular substratum linking developmental susceptibility to late-life neurodegenerative disease. From this perspective, rather than introducing novel pathogenic mechanisms, aging unmasks and amplifies developmentally programmed metabolic and neuroimmune liabilities, completing the second hit in a life-course trajectory toward neurodegeneration.

### Translational implications: targeting α7nAChR signaling and fructose metabolism to modify latent vulnerability across the lifespan

α7nAChRs emerge as strategic molecular nodes linking early environmental programming to late-life neurodegenerative outcomes. Beyond their anti-inflammatory role (118, 119), α7nAChRs function as metabolic and membrane-sensitive biosensors, whose activity is critically shaped by lipid composition, cholesterol distribution, and cellular energetic state across the lifespan (126–128). Rather than reflecting late-stage pathology alone, α7nAChR dysregulation integrates metabolic, inflammatory, and synaptic signals across development, adulthood, and aging, thereby shaping long-term brain resilience or susceptibility to disease conditions (85, 129).

Cumulative metabolic stresses along aging progressively disrupt α7nAChR–dependent signaling, contributing to mitochondrial decline, vascular damage, cholesterol dysregulation, and chronic neuroinflammation. The initial reduction of α7nAChR function exacerbates microglial activation, destabilizes synaptic energy balance, and accelerates neurodegenerative cascades, thereby translating early-life susceptibility into overt pathology. From a translational standpoint, this framework shifts the focus from late-stage neurodegenerative pathology to earlier, modifiable proneness status. In this scenario, α7nAChRs hold promise as integrative biomarkers reflecting the combined status of cholinergic signaling and metabolic health. When affected by pathology, they serve as valuable markers of neuroinflammatory tone. Hence, interventions aimed at preserving or restoring α7nAChR functionality through pharmacological intervention, nutritional strategies, or metabolic optimization may attenuate both neurodevelopmental alterations and age-related neurodegenerative risk (83, 85, 130–134).

Interventions aimed at preserving α7nAChR signaling, reducing fructose exposure, pharmacologically inhibiting GLUT5 or KHK, or limiting polyol pathway activation may effectively attenuate latent vulnerability before clinical symptoms emerge. Consistent with this view, the beneficial effects of metformin and other hypoglycemic agents in Alzheimer disease may result not only from increased insulin secretion and insulin sensitivity, but also from reduced activation of the polyol pathway and diminished endogenous fructose production, thereby contributing to preserving microglial metabolic flexibility and immune function (24, 69, 135–140).

## Conclusions

**Figure 1:**
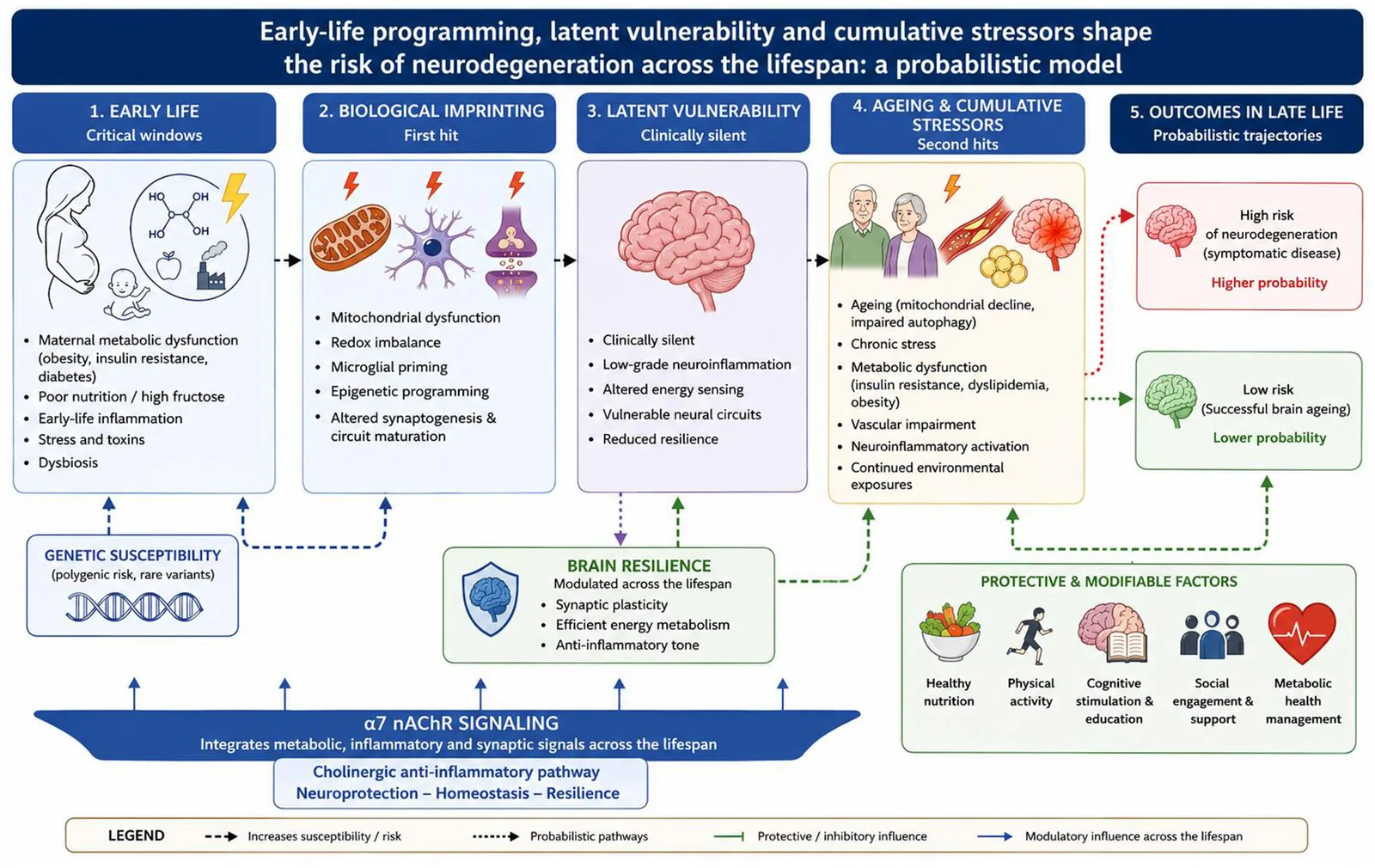
Early-life programming, latent vulnerability, and cumulative stressors shape the risk of neurodegeneration across the lifespan: a probabilistic model. Conceptual model illustrating how early-life metabolic and environmental factors may contribute to biological programming, latent brain vulnerability, and increased risk of neurodegeneration later in life. Genetic susceptibility, brain resilience, protective factors, and α7nAChR signaling may modulate these trajectories across the lifespan.

Neurodevelopmental and neurodegenerative disorders have traditionally been studied as distinct clinical and neuropathological nosological entities. However, growing evidence indicates that they share convergent molecular, metabolic, and inflammatory mechanisms that unfold across the lifespan. By integrating developmental programming, latent vulnerability, and age-related stressors, the two-hit model provides a coherent framework to explain how early-life environmental and metabolic insults shape long-term brain susceptibility to disease conditions. This life-course perspective is associated with the notion that early neurodevelopmental alterations may remain clinically silent for decades, yet establish a primed neuropathological status characterized by impaired energy metabolism, glial dysregulation, and reduced resilience to later insults. Aging, metabolic dysfunction, and cumulative environmental stressors subsequently act as a second hit, unmasking this latent susceptibility and accelerating neurodegenerative processes. Sex-specific metabolic vulnerability may modulate both the intensity and timing of the second hit, contributing to differential disease trajectories between men and women (Figure 1).

Comprehending brain disorders through the perspective of a lifetime continuum offers a unifying framework for neuropathological marker identification, development of early diagnostic tools, prophylactic practices and therapeutic interventions for both neurodevelopmental and neurodegenerative disorders. Whereas addressing direct longitudinal causal evidence in humans often provides a rather limited number of interventional possibilities, converging experimental, epidemiological, and mechanistic data afford a much wider palette of strategies to modify the evolution of dysfunction and transition from neurodevelopmental to neurodegenerative status.

### Future research directions

To move this framework from neuropathological theory to clinical application, future research should prioritize three key areas:

1. Validation of neuropathological markers (141): There is an urgent need for non-invasive neuropathological markers indicative of “latent vulnerability” during the asymptomatic, clinically silent period.

2. Sexual dimorphism in neurometabolism: given the heightened vulnerability of microglia to metabolic collapse in females, particularly for carriers of the *APOE*ε*4* risk allele, research must transition toward sex-specific preventive strategies.

3. The fructose-α7nAChR-lipid axis: Further mechanistic studies are required to elucidate how endogenous fructose production (polyol pathway) directly modulates the localization of the α7nAChR in lipid nanodomains (142), eventually leading to the development of specific pharmacological interventions at these membrane compartments. This calls for a better understanding of site-dependent nAChR pharmacological responses that may lead to the development of new compounds that modulate α7nAChR-mediated anti-inflammatory responses with higher selectivity.
